# Perforated Rectal Cancer Presenting With Symptoms of Sciatic Nerve Compression: A Case Report

**DOI:** 10.1155/cris/8680616

**Published:** 2025-07-22

**Authors:** J. L. O'Sullivan, L. Vu, P. Tan

**Affiliations:** Department of General Surgery, Royal Perth Hospital, Wellington Street, Perth, Washington, Australia

**Keywords:** abscess, colorectal cancer, perforation

## Abstract

In this paper, we discuss the case of a late presentation, locally advanced rectal cancer that has perforated into the greater sciatic notch, presenting with symptoms of lower limb pain and recurrent falls. In this case, we discuss the complexities of diagnosing and managing atypical presentations of colorectal cancer.

## 1. Introduction

Rectal cancer accounts for 30% of colorectal cancer, and the clinical manifestations are highly variable [1]. Perforated colon cancer is an uncommon presentation with an estimated incidence of 3%–10% reported in the literature and is the second most common indication for emergency surgery [2]. Most reported perforation sites include the anterior abdominal wall, inguinal region, and surrounding structures [3, 4]. Perforated colorectal cancer is a poor prognostic indicator with a reduced overall survival [5, 6]. We present a case of the first presentation of a perforated rectal cancer discovered on investigation for recurrent falls.

## 2. Case Report

We present the case of a 73-year-old man who presented to the emergency department with recurrent falls, antalgic gait, and right lower leg weakness. He described a sharp shooting pain and paresthesia radiating down his leg to the foot. In the S1/S2 dermatomal distribution. Neurological exam revealed no atrophy or asymmetry in the affected leg. There was tenderness over the sciatic nerve pathway, particularly around the sciatic notch, with impacted active and passive range of motion at the hip joint. Reflexes and sensation were intact. Bladder control was intact. Straight leg raise test was negative. Antalgic gait was evident on mobilization. The review of systems noted a 10 kg unintentional weight loss, altered bowel habits with predominant constipation, and blood in the stool. He did not have any significant medical, surgical, or family history. There was no history of previous investigations, including a colonoscopy. He is a smoker with a 60-pack-year history and has an alcohol intake of 10 units a night.

**Figure 1:**
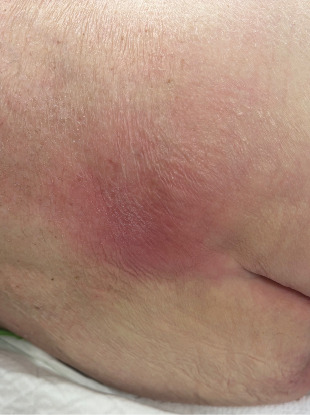
Large 10 × 10 cm area of induration and erythema of the right sacral/gluteal region that was tender to palpation.

**Figure 2:**
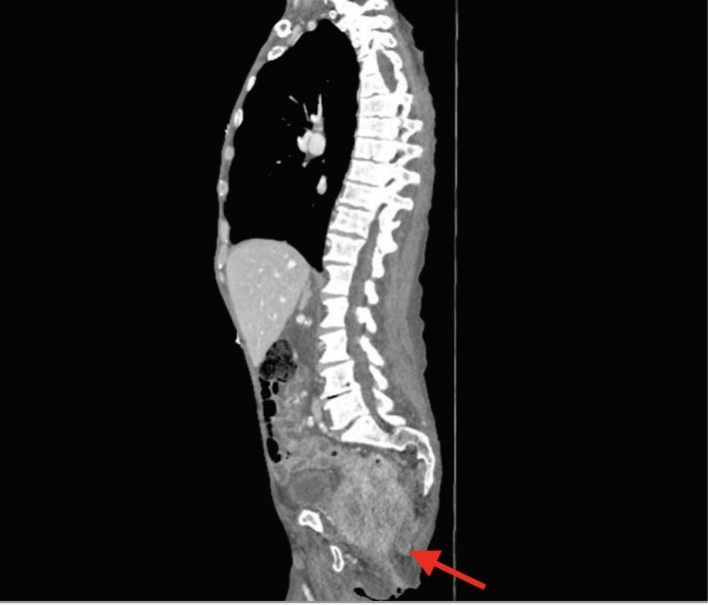
CT of the abdomen and pelvis illustrated a mass in the sigmoid colon extending into the rectum that invaded the lower sacrum, coccyx, mesorectal fat, and pelvic side walls.

**Figure 3:**
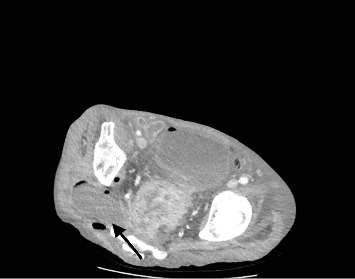
CT of the abdomen showing multiple gas-containing collections in the right gluteal region with the largest one extending into the greater sciatic notch measuring 8 × 4.6 cm.

Examination revealed a cachectic male with a mildly distended abdomen and bilateral palpable groin nodes. Further examination revealed a pressure sore over the sacrum and a large 10 × 10 cm area of induration and erythema of the right gluteal region that was tender to palpation. (Figure 1). PR examination demonstrated a near obstructing, fixed, circumferential rectal mass at 6 cm from the anal verge with purulent/feculent discharge per rectum, and satisfactory rectal tone. Laboratory tests revealed a high white cell and CRP, hemoglobin of 60 g/dL, and a CEA of 10. A CT of the abdomen and pelvis illustrated a large mass in the sigmoid colon extending into the rectum with invasion into the lower sacrum, coccyx, mesorectal fat, and pelvic side walls (Figure 2). There were multiple gas-containing collections in the right gluteal region, with the largest one extending into greater sciatic notch measuring 8 × 4.6 cm (Figure 3). There was intensive mesorectal lymph node involvement and bilobar liver metastases.

## 3. Management

The patient was referred to a specialist colorectal service. Initial treatment included examination under anesthesia, with incision and drainage of the right gluteal abscess. Intraoperatively, the right gluteal cavity was extended deep to the gluteal muscles and into the pararectal space, with frank pus discharging from the incision. A soft 26F chest drain was inserted and secured for ongoing drainage of the space. Wound swabs were sent for microbial sensitivities. The patient was commenced on broad-spectrum antimicrobials.

Repeat neurological examination revealed almost completely resolved antalgic gait, with reduced symptomatology. Unfortunately, the patient developed sepsis postoperatively. Septic screen showed blood cultures positive for E.coli. Appropriate antimicrobial therapy was commenced, and repeat CT abdominal imaging revealed residual pelvic collections not being drained by the chest drain, prompting the placement of a radiologically guided pigtail drain for drainage of the collections and effective sepsis management

Further investigation and diagnostic workup included a flexible sigmoidoscopy, which revealed a malignant-looking mass that could not be transversed with the sigmoidoscope. Biopsies were taken and sent for histopathology. The histopathology demonstrated a low-grade adenocarcinoma MMR proficient, BRAF V600E wildtype, with no microsatellite instability.

**Figure 4:**
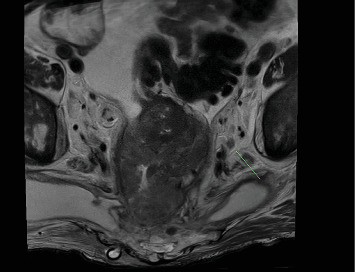
MRI Pelvis demonstrating the rectal tumor with complete invasion of the mesorectal plane, occupying the entirety of the muscularis propria and mesorectal fat.

Further workup included a CT Chest for staging, MRI pelvis, and PET scan. These investigations diagnosed a T4N2M1 rectal tumor with complete invasion of the mesorectal plane, occupying the entirety of the muscularis propria and mesorectal fat with lung, liver, and bone metastases (Figure 4).

The patient was discussed in a multidisciplinary meeting with a treatment plan for palliative chemotherapy and radiotherapy. Nonoperative management was indicated due to the presence of significantly advanced disease with multiple sites of metastases. However, due to concerns regarding potential large bowel obstruction, the patient underwent formation of a loop colostomy as a palliative measure.

Due to the prolonged hospital stay and significantly advanced disease the patient experienced functional decline, and the burden of liver disease ultimately led to hospice care prior to initiation of therapy.

## 4. Discussion

We present a case of advanced, perforated rectosigmoid cancer, which has extended through the greater sciatic notch, resulting in a large right-sided gluteal abscess, causing extrinsic compression of the sciatic nerve, which results in right lower leg pain, paraesthesia, antalgic gate, and weakness, leading to recurrent falls. This case highlights the multiple challenges in the late presentation of a locally advanced, perforated, and metastatic rectal cancer, and the modalities in which an abscess may spread. The patient's symptoms largely resolve after drainage of the abscess, showing the mass effect that the abscess in the greater sciatic notch was having on the nerve. This presentation has not been described previously in the literature.

Two reports were identified with similar pathology and presentations. The case presented by Highton et al. [7] describes an atypical presentation of perforated rectal carcinoma- a patient presenting with lower leg pain and a collection of the posterior thigh that developed near the piriformis muscle. Here, the sciatic foramen is serving as a conduit for abscess infiltration of the soft tissues surrounding it, potentially impinging the sciatic nerve.

A second case describes a man presenting with symptoms of a deep venous thrombus (DVT) of the thigh. On investigation, imaging revealed subcutaneous gas, and a rectal exam uncovered a perforated rectal malignancy. Gas and infection tracked via the sciatic foramen into thigh compartments, causing a localized inflammatory response [8].

Nerve involvement in pelvic malignancies occurs via a few different mechanisms. The most common is direct nerve involvement via tumor invasion or compression [9]. This is seen in malignant tumors of the pelvis, including rectal cancer. These may directly compress the sciatic nerve and result in pain, sensory, and motor deficits in the sciatic nerve distribution. Nerve sheath tumors, lipomas, or schwannomas may compress the nerve itself in a similar manner causing symptoms [10].

Perineural spread is another mechanism of nerve involvement in pelvic malignancy. Tumor cells migrate along the nerve sheaths, which may lead to lumbosacral plexopathy or sciatic neuropathy, and is reported most commonly in gynaecological cancers [11].

As in the above case, secondary inflammatory or infectious processes can also cause symptoms and mass effect due to the formation of necrosis, abscesses or even by severe inflammation. In our case, the piriformis–sciatic foramen connection allows retroperitoneal perforation to propagate posteriorly toward the thigh and buttock muscles, where the sciatic nerve resides.

This case illustrates the diagnostic challenges posed by atypical presentations, such as neurological symptoms overshadowing abdominal complaints. This often leads to a misdiagnosis or delayed diagnosis. Delays caused by atypical presentations often leads to more advanced stage disease at diagnosis, limiting treatment options and worsening prognosis. This also lead to higher rates of complications and reduced overall survival [12, 13].

Early recognition and imaging (e.g., CT or MRI) can help identify the source of the patient's symptoms and guide appropriate surgical and medical interventions. The literature has shown that prognosis is worse for patients who commence treatment more than 45 days after diagnosis, highlighting the importance of timely intervention [12].

One must carefully consider the detailed management strategies and decision-making processes involved in navigating complex cancer cases with a multidisciplinary approach. This includes discussion at multidisciplinary colorectal cancer meetings, ensuring coordination of care, streamlining the diagnostic processes, optimizing treatment planning, and addressing the oncologic and nononcologic aspects of care. Involvement of the multidisciplinary team early in treatment has been shown to improve diagnostic accuracy and enhance overall patient outcomes [14]. This collaborative approach must have patient and family involvement at all stages of care.

## Data Availability

All data relevant to this case report are included within the article. Due to the nature of this case report, no additional datasets were generated or analysed. Patient-related clinical details and images have been anonymized to protect patient privacy, in accordance with ethical standards.
